# A mobile lab for ancient DNA extraction in Peru

**DOI:** 10.6026/973206300181114

**Published:** 2022-12-31

**Authors:** Luis Jaramillo-Valverde, Andrés Vásquez-Domínguez, Kelly S Levano, Rony Castrejon-Cabanillas, Pedro Novoa-Bellota, Marco Machacuay-Romero, Ruth Garcia-de-la-Guarda, Raul J Cano, Ruth Shady Solis, Heinner Guio1

**Affiliations:** 1 ALBIOTEC, Lima, Peru; 2 INBIOMEDIC Research and Technological Center, Lima, Peru; 3 Faculty of Health Sciences, Universidad de Huánuco, Huánuco, Peru; 4 Faculty of Biological Sciences, Universidad Nacional Mayor de San Marcos, Av. Venezuela Cdra 34 S/N, Ciudad Universitaria, Lima 01, Lima, Peru; 5 Helene Fuld College of Nursing, New York, USA; 6 Zona Arqueológica Caral, Unidad Ejecutora 003, Ministerio de Cultura., Lima, Peru; 7 Professional School of Archaeology, Faculty of Social Sciences, Universidad Nacional Mayor de San Marcos, Lima, Perú; 8 Laboratory of Molecular Microbiology and Biotechnology, Faculty of Biological Sciences, Universidad Nacional Mayor de San Marcos, Lima, Perú; 9 The BioCollective, Denver, CO 80014, USA; 10 Centro de Investigación en Biodiversidad para la Salud, Universidad Privada Norbert Wiener, Lima, Perú

**Keywords:** Ancient DNA, mobile laboratory, archeological samples, coprolites

## Abstract

We report the use of a mobile laboratory set up to extract ancient DNA (aDNA) from 34 human coprolites (fossilized faeces) samples. Our approach enabled the rapid genetic characterization of 5,000 years old archeological samples. It is useful for the on-site screening of museums and freshly excavated samples for DNA. This approach is accessible to other investigators as the mobile laboratory was set up using commercially available instruments.

## Background:

Since 2007, the field of ancient DNA (aDNA) has experienced deep changes that come both from technological advances and the attention paid to a variety of DNA sources. The development of next generation sequencing (NGS) has revolutionized ancient DNA research like almost no other field of genetics. Within a few months of the introduction of NGS in 2005 [1,2] published 13 million bp from the nuclear genome of the extinct woolly mammoth. As a complementary approach, new experimental procedures for DNA extraction [3,4] or sampling [5,6] have been described. Until a few years ago, very few studies investigating functional genetics from ancient samples have been conducted and all of these have used conventional cloning and Sanger sequencing [7,8]. The capability to sequence entire genomes from ancient samples now makes it feasible to obtain a large amount of functionally informative nuclear data from sub fossil remains and, as a result, opens up huge potential for future ancient DNA studies. As is widely known, retrieval of ancient DNA can be challenging due to DNA degradation, chemical damage in the DNA molecules leading to incorrect DNA identification and contamination with modern DNA. To target contamination, a number of authenticity criteria for ancient DNA sequence data (including Sanger and NGS data) have been suggested, including the use of a dedicated ancient DNA clean room facility for all pre-amplification work with ancient DNA [9,10, 11]. The importance of using such a clean room not only in the process of DNA extraction, but in subsequent analysis as well, was demonstrated in 2006, when two studies focusing on nuclear DNA from the Neanderthal sample produced inconsistent results [12, [Bibr R13] 14]. It was shown that DNA contamination occurred during the subsequent library preparation for NGS, which was carried out in a non-clean laboratory after DNA extraction [12]. The ancient extract can also contain significant amounts of contaminating human DNA, especially if the sample was collected and handled without DNA studies in mind, as is common for most museum specimens. Thus, all of the issues of between-laboratory contamination control precautions, as well as the pre-laboratory contamination precautions, are even more important than they were in pre-NGS days. Another challenge is the time it takes from sample collection to the delivery of DNA data (days-weeks). Many studies are paying great attention to on-field rapid DNA analysis. They are being standardized for medical diagnosis at the point-of-care, food testing, detection of bio threat agents, to name a few [15, 16]. Progress accomplished thanks to such studies together with the increasing need to avoid the dissemination of museum collections and the observation that freshly excavated fossils are best for ancient DNA analysis [17,18] call for the evaluation of the on-field approach for ancient DNA studies. As more and more researchers become interested in ancient DNA studies, there is an increased need for suitable facilities in which to conduct these studies. Authenticity requirements for ancient DNA data have been proposed previously [9,10,11] and guidelines for work with ancient DNA always include the requirement of a dedicated, isolated laboratory environment [9]. Through the years, different sets of guidelines have defined a suitable ancient DNA work space, however many of them have been established as a result of personal and unpublished experiences. Knowing the major challenge contamination with modern or previously amplified DNA presents to ancient DNA analysis, we attempted to obtain the proof-of-concept for an ancient DNA mobile laboratory by focusing on DNA from coprolites of ancient inhabitants of the Caral Civilization extinct species or species no longer present in the locations where our studies were performed. We report here on the use of a mobile laboratory consisting of commercially available devices for DNA extraction. We conducted studies on samples from 6 zones of Caral Civilization (Figure 1-see PDF).

## Material and Methods:

## Technical requirements for ancient DNA mobile laboratory:

Contaminating DNA can be introduced to an experiment in multiple ways, including through contaminated reagents or samples and through carry-over of DNA from previous experiments [19]. Standard precautions to achieve this include:

## Location of facility:

Spatial isolation of the ancient DNA facility from the post-PCR laboratory is essential. Many established ancient DNA research groups go as far as to have the ancient DNA facility in a separate building from any post-PCR laboratories. Separate access to the ancient DNA facility is ideal. It is established practice to only access the aDNA facility first thing in the day.

## Design:

Ideally, different steps can be conducted in dedicated hoods. While the setup may vary between different ancient DNA facilities depending on the space available, we recommend a minimum of two separate rooms. This allows for the separation of two major working areas allowing for specific activities:

## Room 1:

Changing into dedicated clean room clothing (such as coveralls, hairnet, facemask, laboratory shoes, double gloves), storage of consumables, ultraviolet (UV) C irradiation of samples and consumables.

## Room 2:

DNA extraction and manipulation setup, ideally in separate hoods fitted with internal UVC Spatial limitations may require alternative arrangements. In (Figure 2-see PDF) we describe an example setup for a two-room ancient DNA facility.

## Access:

To reduce contamination, aDNA research groups often practice a limited access policy. Access to the facility should be limited to trained personnel and maintenance staff who understand the protocols for reducing potential contamination. For this reason, some aDNA facilities are fitted with windows to allow guests to view work being undertaken in the laboratory. Such windows also can be an additional safety feature for staff working alone in the laboratory.

## Consumables and equipment:

In addition to standard molecular laboratory equipment, a few more items are advantageous in the aDNA facility. To keep levels of environmental DNA low, the aDNA facility can be UV irradiated when it is not in use. For this purpose, UVC light (= 254 nm) is often used and overhead UV lighting of the lab is ideal. It should be noted that both bleach and UVC can be damaging to some surfaces, a factor that should be taken into account when building the aDNA facility; use bleach- and UVC- resistant materials wherever possible. Further useful features are a positive pressure systems and HEPA-filtered air conditioning. Dedicated laminar flow hoods and fume hoods for DNA extraction and manipulation further reduce the risk of contaminating the experiment.

## Laboratory protocol:

Laboratory protocols vary and depend on the organisms on which research is being undertaken. This is not only valid when human DNA is targeted but in particular also when human associated bacterial DNA is investigated. However, for NGS studies even non-target DNA can become a nuisance as it will get sequenced along with the target DNA and reduce the sequence reads on target. It is therefore recommended to reduce the amount of DNA in the ancient DNA facility as much as possible. Dedicated clean room clothing such as full body c8overalls (as are routinely used in forensic work) can help achieve this goal. Wearing face masks, faceshields and hairnets further reduces the amount of DNA shed by the researcher. Dedicated clean room shoes, wearing two pairs of gloves and regular changing of gloves are useful to reduce carry-over contamination.

## Archaeological specimens:

Arrival at the Archaeological Zone of Caral (Figure 1-see PDF), began with the limitation of the areas where the samples were to be collected (Figure 2). Each area to be sampled was delimited by a yellow tape to prevent the traffic of the other people. Six zones of the Caral Archaeological Zone were chosen. Collecting a total of 34 samples of coprolites in good condition helped us to extract their genetic material (Figure 3-see PDF).

## Overview of experimental procedures:

The equipment used for DNA studies was carried to archeological site in the luggage compartment of a mid-size car. In addition to the machines and kits described below that were used for DNA extraction and analysis, small equipment and consumables consisted of tubes, pipettes, pipette filter tips, disposable clothes, surgical blades, Petri dishes, aluminum foil, bench coat protector, biohazard bags, buffers and ultrapure distilled water. The full list of laboratory material is available from the authors. Temperature-sensitive reagents were transported in a cool box and stored at -20°C upon arrival. Investigators responsible for DNA studies (LJV, AVD) wore masks, hair nets, disposable lab coats and gloves. Working surfaces were covered with versi-dry protection paper sheets that were changed after each experiment. All material related to the dissection of archeological samples was changed between samples. There was no post-DNA extraction of the samples in archeological site. Wastes were brought back to the laboratory.

## DNA extraction:

Prior to DNA extraction, samples were prepared by discarding the outermost portions of the coprolite samples to eliminate risk of contamination, and a replica of each sample was obtained for further analysis (Figure 4-see PDF). This amount of material corresponds to 250 - 300 mg. Ancient genomic DNA was extracted using the PowerSoil DNA Isolation Kit from Qiagen, following the manufacturer's instructions [20].

## DNA sequencing:

The V4 variable region of bacterial 16S rRNA genes will be targeted for high throughput sequencing using predetermined primers 515F (GTGYCAGCMGCCGCGGTAA) and 806R (GGACTACNVGGGTWTCTAAT). In addition, for genetic identification of fungi, ITS analysis will be performed using primers ITS1 (CTTGGTCATTTAGAGGAAGTAA) and ITS4 (GCTGCGTTCTTCATCGATGC) [21]. Library construction and sequencing will be performed in MR-DNA laboratory (www.mrdnalab.com, Shallowater, TX, USA), and sequences will be determined using an Illumina MiSeq instrument following the manufacturer's guidelines. Barcodes for sequences <150 bp, sequences with ambiguous bases, and homopolymer runs greater than 6 bp were removed using the open access mrDNA free software [22].

## Results:

## Mobile DNA laboratory:

The archaeologist's house in Caral includes a room for archiving archeological and paleontological remains excavated from the ancient civilization, and where we installed our mobile laboratory. We analysed 34 coprolites samples originating from six zones of ancient Civilization. (Figure 5-see PDF) shows the mobile DNA laboratory and key steps for DNA studies. The entire process, from archeological samples to DNA data, can be performed within 6 days: a 72h incubation time, a 2-h extraction step. Our device allows DNA extraction from 02 samples (including mock extracts) at a time.

## Analysis of DNA coprolites samples:

The libraries were prepared using Nextera DNA Flex library preparation kit (Illumina) following the manufacturer's user guide. Samples were cleaned using DNEasy PowerClean Pro Cleanup Kit (Qiagen) followed by whole genome amplification by using REPLI-g Midi kit (Qiagen). The linear amplified DNA were cleaned using DNEasy PowerClean Pro Cleanup Kit (Qiagen) and concentrations were evaluated (Table 1-see PDF) using the Qubit® dsDNA HS Assay Kit (Life Technologies).

## Discussion:

This work evaluated the feasibility of using a mobile platform to perform ancient DNA analysis in museums lacking facilities for genetic studies, and as a complementary approach to archeological fieldwork during an excavation campaign. Successful concentration DNA analysis was achieved for several coprolites' samples from human. Several lines of evidence support the notion that our method provides reliable data, notably that contamination and specificity issues were adequately addressed [23]. Our mobile platform consists of three devices for DNA extraction (water bath, mini centrifuge, and vortex). In order to make easily accessible the on-field approach, we paid special attention to set-up a platform that only includes commercially available devices. The full set of instruments and pipettes of our mobile platform amounts to 20,000$, and the cost to perform DNA extraction is 15$. The methods described here allow a single investigator to perform DNA extraction. The procedures used for sample preparation and DNA extraction were designed to limit the working time and the number of instruments in the platform. The samples we screened originate from sites in Caral that have not been studied previously for ancient DNA. In Caral, we focused on coprolites samples from humans that inhabited 5,000 years ago.

## Conclusion:

The development of NGS technology has created tremendous new opportunities for ancient DNA research. As a result, an increasing number of researchers are establishing ancient DNA facilities. We also suggest that modern DNA labs using NGS for environmental s equencing might want to consider some of the issues raised here ascontamination is a problem not limited to ancient DNA alone. This work demonstrates the feasibility to perform ancient DNA analysis under a variety of working condition, including nearby an excavation site. With the ongoing progress of methods for DNA extraction, we anticipate great interest in the near future for on-field ancient studies.
